# Advances in ethylene signalling: protein complexes at the endoplasmic reticulum membrane

**DOI:** 10.1093/aobpla/pls031

**Published:** 2012-10-31

**Authors:** Chuanli Ju, Caren Chang

**Affiliations:** Department of Cell Biology and Molecular Genetics, University of Maryland, College Park, MD 20742, USA

## Abstract

The gaseous plant hormone ethylene plays critical roles in plant responses to environmental and endogenous signals that modulate growth and development. Over the past 25 years, great progress has been made in elucidating the ethylene signalling pathway. Genetic studies in *Arabidopsis thaliana* have identified key components of the pathway, and subcellular localization studies have shown that most of these components, other than transcription factors and protein turnover machinery, are associated with or lie within the endoplasmic reticulum (ER) membrane. The ethylene receptors are found in high-molecular-mass protein complexes and interact with the CTR1 serine/threonine protein kinase and the genetically downstream EIN2 Nramp-like protein. To more fully understand the ethylene signalling pathway, recent research has focused on examining the molecular connections between these components and how they are regulated. Here, we review recent advances and remaining gaps in our understanding of the early steps in the ethylene signalling pathway taking place at the ER.

## Abstract (precis)

The initial steps in ethylene hormone perception involve proteins that are predominantly localized at the endoplasmic reticulum. This article integrates recent work into a coherent picture of these initial steps and highlights remaining questions.

## Introduction

The plant growth regulator ethylene has long been known to play important roles in many aspects of plant growth and development, including seed germination, seedling growth, flower senescence, fruit ripening, abscission and senescence, and responses to biotic and abiotic stresses (Abeles *et al.* 1992). Ethylene is produced by most plant tissues and cell types, and the biosynthesis of ethylene is tightly regulated both by internal signals and in response to diverse environmental stresses (Abeles *et al.* 1992; Bleecker and Kende 2000; Argueso *et al.*, 2007).

Significant progress has been made in our understanding of how plants perceive and transduce the ethylene signal (e.g. Bleecker and Kende 2000; Stepanova and Alonso 2009). The genetic components of the signalling pathway were first identified by molecular genetic dissection of the ethylene response in *Arabidopsis*. This involved the isolation of ethylene response mutants, such as ethylene-insensitive or constitutive ethylene-response mutants, followed by the molecular cloning of the corresponding genes and the determination of their order of action. These approaches have uncovered a pathway that starts with the ethylene receptors and terminates with transcription factors controlling gene expression. The pathway contains a combination of signalling components not previously known to function together. Many of the remaining questions regarding the pathway have to do with the biochemical mechanisms that these components use to transduce the ethylene signal.

Although the mechanisms of signalling have been somewhat elusive, a cellular framework has come into focus, based on the subcellular localization of the components in the pathway, as well as the identification of protein–protein interactions between them. While changes in gene expression are regulated by transcription factors in the nucleus, interestingly, most of the signalling components, including the ethylene receptors, have been found to reside predominantly at the endoplasmic reticulum (ER) membrane (Chen *et al.* 2002; Gao *et al.* 2003; Grefen *et al.* 2008; Bisson *et al.* 2009). The ER as the site of ethylene perception presents a variation on ligand–receptor paradigms, since receptors for most other signals are typically at the plasma membrane or in the nucleus. Physical associations between the ER-membrane-localized components have added an additional layer to the picture. Thus, the current view of the pathway is that critical steps take place at the ER and involve interactions between key players.

In this article, we focus on the current picture of ethylene signalling at the ER membrane. We review recent progress in understanding the membrane-localized ethylene receptor complex and protein–protein interactions, which are critical to elucidating the mechanisms of ethylene signalling. We also discuss the pathway immediately upstream and downstream of the ethylene receptors, including ethylene receptor biogenesis and signalling output by the receptor complex.

### Overview of the ethylene signalling pathway

**Fig. 1:**
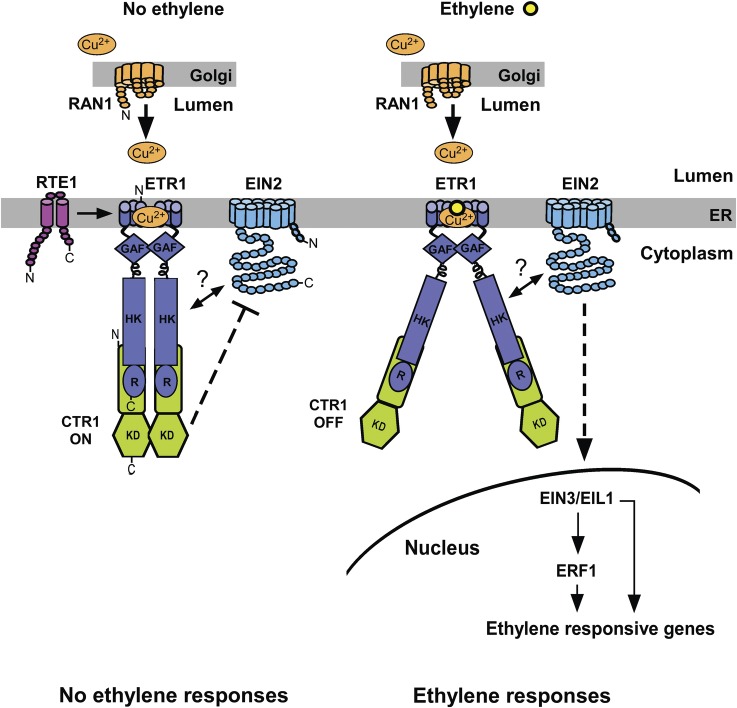
**A model for ethylene signalling at the ER membrane in *Arabidopsis*.** The initial steps in the ethylene signalling pathway occur at the ER membrane and involve ethylene receptors (represented here by ETR1; Chang *et al.* 1993) interacting with the CTR1 serine/threonine protein kinase (Kieber *et al.* 1993) and the EIN2 Nramp homologue (Alonso *et al.* 1999). Left: the N-terminal regulatory domain of the CTR1 protein kinase associates with the ethylene receptor histidine kinase (HK) and receiver (R) domains. In the absence of ethylene, the ethylene receptors activate the CTR1 kinase domain (KD) by an unknown mechanism. Active CTR1 somehow represses EIN2. Right: ethylene binding shuts off receptor signalling, such that the CTR1 kinase domain is no longer active, allowing signalling to proceed to EIN2. We postulate that dimerization/monomerization of CTR1 could play a role in activating/inactivating the CTR1 KD, respectively. The biochemical functions of EIN2 have yet to be determined, but downstream of EIN2 there is activation of the nuclear transcription factors EIN3/EIL1 and ERF1, which induce ethylene-responsive gene expression. Interestingly, EIN2 can also associate with ethylene receptors (Bisson *et al.* 2009; Bisson and Groth 2010), and *in vitro* association of EIN2 with the ETR1 receptor is enhanced when ETR1 histidine kinase activity is disrupted (Bisson and Groth 2010). The P-type ATPase copper transporter, RAN1, provides the copper cofactor required for ethylene binding (Rodriguez *et al.* 1999), and is important for the biogenesis of the receptors (Hirayama *et al.* 1999; Woeste and Kieber 2000; Binder *et al.* 2010), while RTE1 activates the ETR1 receptor by an unknown mechanism (Resnick *et al.* 2006, 2008). The RTE1 membrane topology is unknown and is speculated here.

First we briefly summarize the key players in ethylene signalling and their actions in the pathway (Fig. 1). Ethylene is perceived by a family of ER-membrane-bound receptors that have similarity to the receptor histidine protein kinases of the prokaryotic two-component system (Binder *et al*. 2012). Although some distinct functions have been uncovered (Binder *et al*. 2012), the ethylene receptors are largely functionally redundant. The receptors negatively regulate ethylene signalling, meaning that the receptors repress ethylene responses in the absence of ethylene; mutations knocking out multiple ethylene receptor genes result in constitutive ethylene-response phenotypes, even in the absence of ethylene (Hua and Meyerowitz 1998; Wang *et al.* 2003; Qu *et al.* 2007). The *Arabidopsis* ethylene receptor ERS1, which negatively regulates ethylene responses, has also been found to promote ethylene responses in the presence of the wild-type ETR1 receptor (Liu *et al*. 2010).

The receptors fall into two subfamilies on the basis of phylogenetic relationships and structural similarities. Subfamily I receptors possess histidine autokinase activity, as in the two-component system, whereas the more diverged subfamily II receptors have serine/threonine kinase activity and an additional N-terminal hydrophobic domain, which is a putative signal sequence (Gamble *et al.* 1998; Moussatche and Klee 2004; Chen *et al.* 2009). In *Arabidopsis*, the subfamily I receptors (ETR1 and ERS1) have a predominant role in controlling ethylene responses, but their signalling mechanism is unclear (Hua and Meyerowitz 1998; Qu *et al.* 2007). Genetic studies have indicated that histidine autophosphorylation plays only a minor role in ethylene responses (Wang *et al.* 2003; Hall *et al.* 2012). Recent genetic findings suggest that the binding of ethylene by the *Arabidopsis* ETR1 ethylene receptor stimulates its histidine autokinase activity (Hall *et al.* 2012), whereas an *in vitro* biochemical analysis of ETR1 indicates the opposite—that ethylene binding inhibits such activity (Voet-van-Vormizeele and Groth 2008). Thus, the relationship between ethylene binding and histidine autophosphorylation is not resolved, nor do we understand the primary biochemical mechanism of ethylene receptor signalling.

The CTR1 protein kinase, a negative regulator of ethylene responses (Kieber *et al.* 1993), associates with and acts downstream of the ethylene receptors (Clark *et al.* 1998; Gao *et al*. 2003; Zhong *et al.* 2008). As with the ethylene receptor genes, loss of *CTR1* function confers constitutive ethylene responses. From genetic studies, we know that in the absence of ethylene perception, the receptors repress ethylene responses by activating CTR1; binding of ethylene inactivates ethylene receptor signalling and CTR1 is consequently inactive, thereby leading to ethylene responses (Zhong and Chang 2012) (Fig. 1). The molecular mechanism for how the receptors control CTR1 activity is unclear. CTR1 is most similar in sequence to Raf, a mitogen-activated protein kinase kinase kinase (MAPKKK) (Kieber *et al.* 1993). Thus, CTR1 has been presumed to function, like Raf, in a typical MAPK cascade. Yet there are no MAPKKs/MAPKs that have been conclusively shown to be controlled by CTR1, and reports of such kinases in ethylene signal transduction are controversial and under debate (Ecker 2004; Hahn and Harter 2009; Zhao and Guo 2011).

The next known component downstream of CTR1 is EIN2, a central positive regulator of ethylene responses; loss of *EIN2* function confers complete ethylene insensitivity (Alonso *et al.* 1999). Like the ethylene receptors, EIN2 resides in the ER membrane (Bisson *et al.* 2009). The N-terminus of EIN2, consisting of 12 predicted transmembrane helices, has similarity to N-ramp metal ion transporters. Whether EIN2 transports a metal and how such transport might be connected to ethylene signalling are unknown. The EIN2 C-terminal domain is predominantly hydrophilic and predicted to be cytosolic (Alonso *et al.* 1999). Although EIN2 plays an indispensable role in ethylene response, it is not known how EIN2 is activated at the biochemical level, nor how EIN2 relays the ethylene signal to downstream proteins. EIN2 protein levels are controlled in response to ethylene. In the absence of ethylene, two F-box proteins (ETP1/2) target the EIN2 protein for degradation by the 26S proteasome; the degradation is inhibited in the presence of ethylene, resulting in the accumulation of EIN2 (Qiao *et al*. 2009). Interestingly, EIN2 has been found to interact with all five *Arabidopsis* ethylene receptors (Bisson and Groth 2010), raising the possibility that the ethylene receptors play a role in regulating EIN2 activity.

The next known components in the pathway are found in the nucleus. The two master transcription factors, EIN3 and EIN3-LIKE1 (EIL1) (Chao *et al.* 1997), are both degraded by the 26S proteasome in the absence of ethylene (Guo and Ecker 2003; Potuschak *et al.* 2003; Gagne *et al.* 2004; An *et al*. 2010). EIN3 directly activates expression of the ERF1 transcription factor, which in turn activates the expression of other ethylene-responsive genes (Solano *et al.* 1998).

## Ethylene receptor complexes at the ER membrane

### Ethylene perception at the ER

Plant ethylene receptors have a conserved N-terminal ethylene-binding domain, followed by a GAF domain (c**G**MP-specific phosphodiesterases, **a**denyl cyclases, **f**ormate hydrogen lyase transcriptional activator) and putative signal output motifs (a histidine kinase domain with or without an attached receiver domain) in their C-terminal portions (Gamble *et al.* 1998; Moussatche and Klee 2004; Gao *et al.* 2008). The ethylene-binding domain is comprised of three N-terminal transmembrane domains (Schaller and Bleecker 1995; Hall *et al.* 1999; Rodriguez *et al.* 1999; O'Malley *et al.* 2005), which are also involved in membrane localization and dimerization of the receptors (Rodriguez *et al.* 1999; Chen *et al.* 2002). The subfamily II receptors contain a fourth transmembrane domain, which possibly serves as a signal sequence for membrane targeting.

There is substantial evidence indicating that the ethylene receptors are targeted to the ER membrane, including biochemical fractionation of membranes, immunolectron microscopy and fluorescence tagging (Chen *et al.* 2002, 2007; Ma *et al.* 2006; Dong *et al.* 2008; Grefen *et al.* 2008; Zhong *et al.* 2008). Immunohistochemistry in *Arabidopsis* root hair cells also showed that the ETR1 ethylene receptor can reside at both the ER and Golgi apparatus (Dong *et al.* 2008). In tobacco protoplasts, the tobacco ethylene receptor NTHK1 appears to be at the plasma membrane when overexpressed as a green fluorescent protein (GFP)-tagged version (Xie *et al*. 2003). The primary location of ethylene perception, however, is thought to be the ER endomembrane network.

The ER membrane is not a typical site for receptor–ligand binding, thus raising the question as to why the ER would be the site of ethylene perception. First, it is important to note that as a gaseous hormone, ethylene diffuses into and out of cells, so there is no requirement for the receptors to perceive the ethylene signal at the plasma membrane. Ethylene is also readily diffusible in both aqueous and lipid compartments of the cell, and is actually more soluble within the membrane (Abeles *et al.* 1992). Second, the location of the ethylene receptors might have something to do with their evolutionary history. The ethylene receptor genes originated from the chloroplast genome, and the chloroplast is derived from an ancestral cyanobacterium (Bleecker 1999; Rodriguez *et al.* 1999; Mount and Chang 2002). Sequences from the ancestral ethylene receptor, such as a signal sequence for bacterial export, could be responsible for directing (or mis-directing) the receptors to the ER membrane (Chen *et al*. 2002). Third, it could be advantageous to perceive ethylene at the ER, as suggested by Chen *et al.* (2005). Energetically, it might be more efficient than moving the ethylene receptors through the secretory system to the plasma membrane. It might even allow for a more rapid ethylene response, since the site of receptor synthesis is close to the site of action. In addition to protein synthesis, there are various cellular functions that take place at the ER, including calcium storage, lipid metabolism and stress responses. Conceivably, the ER is also in contact with most other cellular organelles and endomembrane systems, given the network-like structure of the ER. Thus, the localization of the ethylene receptors at the ER might facilitate interactions and integration with cellular responses and other signalling pathways.

### Ethylene receptor homodimers and higher-order clustering

**Fig. 2:**
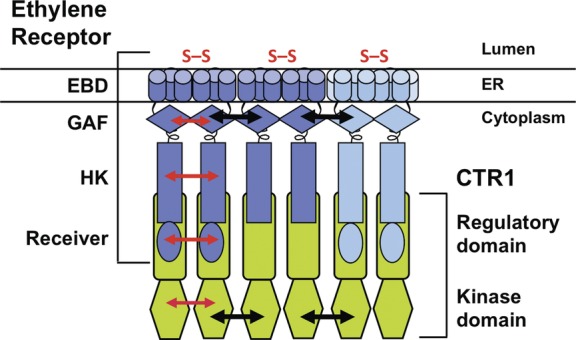
**Model of an active heteromeric ethylene receptor–CTR1 complex at the ER membrane.** The ethylene receptors are tethered to the ER membrane (Chen *et al.* 2002, 2007; Dong *et al.* 2008; Grefen *et al.* 2008) by the N-terminal ethylene-binding domain (EBD). Representative ethylene receptors of subfamily I (in dark blue) and subfamily II (in light blue) are homodimers (Schaller and Bleecker 1995; Gao *et al.* 2008), each stabilized by a pair of intermolecular N-terminal disulfide bonds (S–S) in the lumen (Schaller *et al.* 1995), as well as likely non-covalent interactions (red arrows, shown only on the leftmost homodimer) between the two-component histidine kinase domains (HK), receiver domains and GAF domains. The receptor homodimers form a higher-order complex with neighbouring receptor dimers, mediated in part by the GAF domain (black arrows) (Schaller *et al*. 1995; Hall *et al*. 2000; Gao *et al*. 2008; Grefen *et al*. 2008). The N-terminal regulatory domain of the CTR1 protein kinase (green) physically associates with the HK and receiver domains of the receptors (Clark *et al.* 1998; Cancel and Larsen 2002; Gao *et al.* 2003; Zhong *et al.* 2008). We speculate that each receptor HK domain associates with one CTR1 molecule. Based on crystal structures, the ETR1 receiver domain (Müller-Dieckmann *et al.* 1999) and the CTR1 kinase domain (Mayerhofer *et al.* 2012) are each dimers (red arrows). The CTR1 kinase domain is believed to be active when dimerized (Mayerhofer *et al.* 2012). Moreover, oligomerization of the CTR1 kinase domain dimers (black arrows) may help to bring the ethylene receptors together, reinforcing the receptor complex (Mayerhofer *et al.* 2012). The receptors have also been found in high-molecular-mass complexes containing unidentified proteins (Chen *et al.* 2010) (not shown). The higher-order ethylene receptor complexes may serve to amplify the signal.

The basic unit of the ethylene receptor is a homodimer that is capable of binding ethylene (Schaller and Bleecker 1995). There is also the possibility that heterodimers may form (Gao *et al.* 2008). Two N-terminal disulfide bonds stabilize the dimer (Schaller *et al.* 1995; Hall *et al*. 2000). The receiver domain, possessed by some ethylene receptors, might contribute to dimerization since the ETR1 receiver domain was found to homodimerize in solution (Müller-Dieckmann *et al.* 1999). In *Arabidopsis*, the ethylene receptor homodimers have also been found to form non-covalent higher-order homomeric and heteromeric complexes with each other, mediated in part by the receptor GAF domain (Gao *et al.* 2008; Grefen *et al.* 2008; Chen *et al.* 2009) (Fig. 2). Such plant ethylene receptor clusters might be analogous to the clusters of bacterial histidine kinase-linked chemoreceptors (Baker *et al*. 2006). The higher-order interactions between the ethylene receptors may allow for ethylene receptor signalling conformations to be propagated and amplified by lateral interactions, which might explain how plants can display such a high sensitivity for ethylene.

Fast protein liquid chromatography (FPLC) gel filtration chromatography of *Arabidopsis* ethylene receptors further revealed that the receptors exist in high-molecular-mass protein complexes that are stabilized by disulfide linkages (Chen *et al.* 2010). For example, the ETR1 homodimer is 150 kDa, but is found in a large 750-kDa complex. Deletion analysis indicated that the ETR1 receptor has multiple binding sites for proteins in the complex (Chen *et al.* 2010). The different ethylene receptor isoforms form protein complexes of differing sizes, and the identities of all the proteins in the complexes are not yet known. Candidates include a tetratricopeptide repeat protein TPR1 (Lin *et al.* 2008, 2009) and ECIP1, which interacts with EIN2 (Lei *et al.* 2011). Interestingly, CTR1 and EIN2 do not appear to be in the ETR1 complex, despite their ability to interact with ETR1. The high degree of heterogeneity in the complexes might reflect specificities for particular cellular environments or certain responses. Interestingly, the ERS1 ethylene receptor complex increases in size upon ethylene treatment (Chen *et al.* 2010); such an increase might involve the recruitment of additional proteins into the complex, perhaps depending on the phosphorylation status of the receptor.

## Biogenesis of the ethylene receptors at the membrane

### Role of the copper transporter, RESPONSIVE TO ANTAGONIST (RAN1)

*RAN1* encodes a P-type ATPase copper transporter homologous to the mammalian Menkes/Wilson proteins (Hirayama *et al*. 1999). The *ran1* null mutant has a severe constitutive ethylene-response phenotype in the seedling, similar to that displayed by mutants lacking multiple ethylene receptors, indicating that RAN1 is required for ethylene receptor function (Woeste and Kieber 2000). RAN1 appears to be critical in providing the copper cofactor that the ethylene receptors require for the binding of ethylene (Rodriguez *et al.* 1999; Binder *et al*. 2010). For example, plants lacking *RAN1* consequently lack ethylene-binding ability; moreover, when the ETR1 ethylene receptor is expressed in a yeast cell-based system lacking Ccc2 (the yeast RAN1 homologue), the ability of ETR1 to bind ethylene is restored by the addition of copper ions (Binder *et al*. 2010). By western blotting, the ETR1 ethylene receptor is still detected at normal levels in the membrane fraction of the *ran1* mutant (Zhao *et al*. 2002), suggesting that the lack of function could be due to misfolding. Thus, the copper ion is apparently essential for the biogenesis of the ethylene receptors, as concluded by Binder *et al*. (2010). Interestingly, weaker missense mutations of *ran1* have been found to alter the ligand specificity of the ethylene receptor, rendering the plant capable of responding to the ethylene antagonist trans-cyclooctene (Hirayama *et al*. 1999).

Consistent with the subcellular localization of the mammalian Menkes/Wilson homologues (Camakaris *et al.* 1999), *Arabidopsis* RAN1 has been found at the Golgi apparatus (Dunkley *et al*. 2006). Whether a fraction of RAN1 proteins can also reside at the ER remains an open question. If RAN1 is specifically localized to the Golgi apparatus, then this raises the question of how the ethylene receptors at the ER are able to obtain the copper cofactor. Perhaps there is an unidentified copper chaperone that specifically delivers copper to the ethylene receptors. Alternatively, the nascent ethylene receptors might obtain copper at the Golgi apparatus and then undergo retrograde transport to the ER. Interestingly, ETR1 (expressed under the native ETR1 promoter) was localized to both the ER and Golgi apparatus in *Arabidopsis* root hair cells using immunohistochemistry (Dong *et al*. 2008). Another possibility is that copper delivery to proteins in the secretory compartment is not subjected to tight regulation, as suggested by Binder *et al*. (2010).

### Role of REVERSION-TO-ETHYLENE SENSITIVITY1 (RTE1), a novel membrane protein

One of the *Arabidopsis* ethylene receptors, ETR1, is dependent on an additional upstream component known as RTE1. *RTE1*, also identified as the *Green-ripe* gene in tomato (Barry and Giovannoni 2006), was obtained in a genetic screen for suppressors of ethylene insensitivity conferred by the *etr1-2* dominant (gain-of-function) mutant (Resnick *et al.* 2006). The loss of *rte1* results in a phenotype similar to that of the *etr1* null mutant (Resnick *et al*. 2006; Zhou *et al*. 2007), yet the ETR1 protein is present at normal levels when RTE1 is absent (Resnick *et al.* 2008), suggesting that RTE1 is important for ETR1 folding or activity. RTE1 homologues are found not only in plants, but also in animals. *RTE1* encodes a novel membrane protein that co-localizes with ETR1 at the ER and Golgi apparatus (Dong *et al*. 2008), and can physically associate with ETR1 (Dong *et al*. 2010). The molecular function of RTE1 is unknown but appears to be unrelated to copper transport (Resnick *et al*. 2008; C. Chang, unpublished data). *RTE1* has been proposed to be involved in promoting either ETR1 folding or stabilization of the ETR1 active conformation (Resnick *et al*. 2006, 2008; Zhou *et al*. 2007; Dong *et al*. 2008). Interestingly, only ETR1 and not the other ethylene receptors is dependent on RTE1, despite all of the receptors being in the same protein complex.

## The ethylene receptor–CTR1 signalling complex

### Association of the CTR1 protein kinase with the ethylene receptors

As mentioned earlier, CTR1 is a serine/threonine protein kinase most similar in sequence to the Raf protein kinase family. CTR1 has a novel N-terminal domain, which is presumed to be a regulatory domain, and a conserved C-terminal kinase domain. *In vitro* biochemical studies have confirmed that the *Arabidopsis* CTR1 kinase domain has intrinsic serine/threonine protein kinase activity similar to Raf-1 in enzymatic properties; mutations in conserved residues of the kinase domain disrupt this activity and confer constitutive ethylene-response phenotypes (Huang *et al.* 2003). CTR1 displays intermolecular autophosphorylation *in vitro* (Huang *et al.* 2003). In addition, X-ray crystallography has revealed that the active CTR1 kinase domain forms dimers, whereas an inactive form is a monomer (Mayerhofer *et al.* 2012). Thus, the activation of CTR1 might have parallels with B-RAF activation, which is dependent on dimerization (Rajakulendran *et al.* 2009).

Although CTR1 contains no predicted transmembrane domains (Kieber *et al.* 1993; Huang *et al.* 2003), CTR1 is found at the ER membrane due to its association with the ethylene receptors (Clark *et al.* 1998; Gao *et al.* 2003; Zhong *et al.* 2008). The receptors are believed to activate CTR1 through this interaction, and membrane recruitment might also place CTR1 in contact with other regulatory elements. The N-terminal regulatory domain of CTR1 interacts with the receptor histidine kinase/receiver domain of the subfamily I ethylene receptors in *Arabidopsis* (Clark *et al.* 1998). A similar interaction was shown for the CTR proteins and ethylene receptors of tomato (Lin *et al.* 2008; Zhong *et al.* 2008). *Arabidopsis* CTR1 may have a weaker association with the subfamily II receptor ETR2 in comparison with subfamily I receptors (Cancel and Larsen 2002), but *in vivo* analyses have indicated that CTR1 interacts with all members of the receptor family (Gao *et al.* 2003). Interestingly, the recent crystal structure of the CTR1 kinase domain revealed that it has an allosteric dimer interface, suggesting the oligomerization of CTR1 kinase domain dimers (Mayerhofer *et al.* 2012) (Fig. 2). Mayerhofer *et al.* (2012) thus propose that the interaction of CTR1 dimers with the ethylene receptor dimers reinforces the receptor complex by promoting associations between neighbouring ethylene receptors (Mayerhofer *et al.* 2012).

### Regulation of CTR1 by the ethylene receptors

What is unclear is the molecular mechanism by which the receptors control CTR1 kinase activity. The ethylene receptor–CTR1 association represents a novel combination of proteins that do not fit the existing paradigms for either the Raf-like CTR1 or the two-component receptors. Raf protein kinases are activated by the small GTP-binding protein Ras (Wellbrock *et al.* 2004), whereas histidine receptor kinases typically signal by a multi-step histidine-to-aspartate phosphorelay (Schaller *et al*. 2011). Interestingly, the crystal structures of the ETR1 receiver domain (Müller-Dieckmann *et al.* 1999) and bacterial receiver domains (Chen *et al.* 1990) are similar to that of Ras, which also shows functional similarities with receiver domains (Lukat *et al.* 1991). Thus the interaction between the ethylene receptor's receiver domain and the N-terminal domain of CTR1 might be structurally and functionally analogous to the Ras–Raf paradigm. The interaction of the CTR1 N-terminal domain with the receptor histidine kinase domain, however, is independent of the receiver domain and represents a novel association.

In the typical multi-step histidine-to-aspartate phosphorelay, there is transfer of the phosphate from the histidine autophosphorylation site on the two-component receptor to a conserved aspartic acid residue in the cognate receiver domain, followed by transfer of the phosphate to a small histidine-containing phosphotransfer protein. Finally, there is transfer of the phosphate to a conserved aspartic acid residue in a response regulator protein, which is typically a transcription factor. The activation of CTR1 is unlikely to involve such a phosphorelay by the receptors, since the prevention of histidine kinase activity in the predominant ethylene receptor, ETR1, has only minor effects on ETR1's ability to signal (Wang *et al*. 2003; Hall *et al.* 2012). A more likely mechanism for CTR1 activation could be that the receptors hold CTR1 in an active conformation in the absence of ethylene. When the receptors bind ethylene and presumably undergo a conformational change, there could be a concomitant alteration in the conformation of CTR1 that turns off the CTR1 kinase activity. It is conceivable that the histidine autophosphorylation induced by ethylene binding, as suggested by Hall *et al.* (2012), plays a role in the conformational change that terminates CTR1 activation. Since structural studies show that the CTR1 kinase domain is a dimer when active (Mayerhofer *et al.* 2012), a conformational change causing monomerization of CTR1 could be a possible mechanism for inactivation of CTR1.

The physical association of CTR1 with the ethylene receptors is critical for the activation of CTR1 kinase activity, as indicated by the *ctr1-8* mutation. *ctr1-8* encodes a G354E substitution in the N-terminal domain of CTR1 and abolishes the interaction of CTR1 with the receptors (Gao *et al.* 2003; Huang *et al.* 2003). Although CTR1-8 has wild-type kinase activity *in vitro*, the mutation in the regulatory domain is correlated with reduced CTR1-8 kinase activity *in vivo*, i.e. the *ctr1-8* mutant has a constitutive ethylene-response phenotype similar to that of the kinase inactive alleles of *ctr1* (Huang *et al.* 2003). Similarly, in mutants lacking multiple ethylene receptors, CTR1 is no longer found at the ER membrane and such mutants display constitutive ethylene-response phenotypes, presumably because the reduced interaction with the receptors causes CTR1 to be inactive (Gao *et al*. 2003; Qu *et al*. 2007). This raises the possibility that the regulation of wild-type CTR1 activity could involve a mechanism in which CTR1 dissociates from the ethylene receptors, but not necessarily from the membrane. Interestingly, ethylene treatment actually causes CTR1 to associate more tightly with the membrane (Gao *et al*. 2003). Other proteins are possibly involved in retaining CTR1 at the ER.

While the main signalling pathway involves CTR1, it is worth pointing out that subtle effects of ethylene receptor signalling might occur via the two-component system's phosphotransfer proteins and response regulators in *Arabidopsis* (known as AHPs and ARRs, respectively). This is based on evidence that the ethylene receptors can interact with AHP proteins (Urao *et al*. 2000; Scharein *et al*. 2008) and that a response regulator, ARR2, might have a role as a positive regulator in modulating ethylene responses downstream of ETR1 (Hass *et al.* 2004; Mason *et al.* 2005). Thus, ethylene receptor signalling through AHPs and ARRs might represent an ethylene response pathway that bypasses CTR1. The existence of an alternative ethylene response pathway is supported by the fact that mutants lacking CTR1 display a residual ethylene response (Larsen and Chang 2001; Huang *et al.* 2003). Additionally, mutants lacking multiple ethylene receptors (e.g. the loss-of-function mutant *etr1-6 etr2-3 ein4-4 ers2-3* and the double-mutant *etr1 ers1*) display a more severe phenotype than the *ctr1* loss-of-function mutant (Hua and Meyerowitz 1998; Hall and Bleecker 2003), suggesting that the receptors can signal through an alternate pathway.

### Substrate of the receptor–CTR1 complex

One of the mysteries in the ethylene signalling pathway has been the identity of the immediate downstream substrate of CTR1. Raf, to which CTR1 has the most similarity, is an MAPKKK that functions in an MAPK signalling cascade. The MAPK signalling cascade is a conserved module of three protein kinases consisting of an MAPKKK, a downstream MAPK kinase (MAPKK) and an MAPK, and such pathways regulate a variety of cellular processes in eukaryotes (Rodriguez *et al*. 2010). Consequently, it has long been thought that an unidentified MAPKK is the target of CTR1 phosphorylation. Although there have been reports of MAPKs in ethylene signal transduction (Novikova *et al.* 2000; Ouaked *et al.* 2003; Yoo *et al.* 2008), no conclusive CTR1-targeted MAPKKs or MAPKs have been identified to date (Hahn and Harter 2009; Zhao and Guo 2011), and the MAPKK and MAPKs that were initially assigned to the ethylene signalling pathway have subsequently been found to regulate ethylene biosynthesis (Liu and Zhang 2004; Joo *et al*. 2008; Xu *et al*. 2008). The membrane recruitment of CTR1 could place CTR1 in contact with the next known downstream component in the pathway, EIN2, although a direct CTR1–EIN2 interaction has yet to be shown.

## Association of EIN2 with the ethylene receptors

### ER localization of EIN2 and interaction with the ethylene receptors

The subcellular localization of the EIN2 protein was unknown for a decade after the *EIN2* gene was cloned. EIN2 has a highly hydrophobic N-terminal domain containing 12 predicted membrane-spanning domains, which were shown to be integrated within the membrane by *in vitro* translation with canine pancreatic microsomes (Alonso *et al.* 1999). For a time, EIN2 was considered to be in the nuclear membrane to allow for a physical connection between EIN2 and the downstream nuclear-localized transcription factors. It was finally shown, however, that EIN2 resides at the ER network via co-expression of GFP-tagged EIN2 and an ER marker protein in tobacco cells by Bisson *et al*. (2009). Thus EIN2 is localized to the same endomembrane system that contains the ethylene receptors and CTR1.

Unexpectedly, EIN2 was shown to be capable of interacting with the ethylene receptors. Interaction between EIN2 and ETR1 was detected by fluorescence resonance energy transfer microscopy using tobacco leaf epidermal cells and by *in vitro* fluorescence titration studies (Bisson *et al.* 2009). The interacting domain of EIN2 is the soluble C-terminal domain, which appears to associate with the histidine kinase portion of ETR1 (Bisson and Groth 2010, 2011). Fluorescence resonance energy transfer was also used to confirm that EIN2 co-localizes and associates with all five *Arabidopsis* ethylene receptors *in planta* (Bisson and Groth 2010).

The coincidental localization of EIN2 with the five ethylene receptors and CTR1 at the ER membrane led Bisson *et al*. (2009) to propose the existence of an ‘ER-borne ternary super-complex’. It is unclear whether the interaction occurs simultaneously and how this could fit with the existing genetic model of ethylene signalling, in which EIN2 acts at or downstream of CTR1, based on genetic epistasis. Bisson and Groth (2010) propose that the receptor–EIN2 association could be involved in either protecting EIN2 from proteasome degradation or promoting its signalling. Direct signalling from the receptors to EIN2 could conceivably represent a signalling bypass of CTR1.

Bisson and Groth (2011) suggested that there might be a dynamic interaction between EIN2 and the ethylene receptors, depending on the phosphorylation state of the receptor kinase domain. When an alanine substitution was used to replace ETR1 His353 (which is the site of *in vitro* autophosphorylation), there was a four-fold increased affinity for EIN2 *in vitro*, whereas a His353Glu substitution (designed to mimic phosphorylation) had no effect on the interaction (Bisson and Groth 2010). Additional data showed a four-fold higher affinity for EIN2 *in vitro* in the presence of cyanide (an ethylene agonist; Bisson and Groth 2012), suggesting a possible link between ethylene binding and formation of the receptor–EIN2 complex. This finding is consistent with the His353Ala substitution, assuming that ETR1 has histidine autokinase activity in the absence of ethylene binding. The biological relevance of these findings is unclear however. One unresolved issue is whether the ethylene signal promotes ETR1 histidine autophosphorylation or inhibits it (Hall *et al.* 2012). Secondly, histidine kinase activity plays only a minor role in ETR1 signalling (Hall and Bleecker 2003; Wang *et al.* 2003; Hall *et al.* 2012).

## Conclusions and forward look

Molecular genetics has uncovered an unusual combination of proteins comprising the early events in the ethylene signalling pathway, and subcellular localization and physical association data have revealed the formation of signalling complexes by these proteins at the ER membrane (Figs 1 and 2). Ethylene signalling involves clusters of ethylene receptor homodimers in high-molecular-mass complexes, as well as association of ethylene receptors with CTR1 and EIN2.

Many remaining unanswered questions in ethylene signalling are centred on the biochemical mechanisms that connect the players in the pathway. How do the ethylene receptors signal to CTR1, how does CTR1 signal to EIN2, what is the mechanism of EIN2 signalling, and how do these ER-localized components connect to the nuclear-localized EIN3 and EIL1 transcription factors? Future work may help to resolve the relationship between ethylene binding and receptor kinase activity, and the potential role of a His to Asp phosphorelay from the receptors. We speculate that monomerization of the CTR1 kinase domain in the receptor complex could play a role in inactivating CTR1. It remains to be seen whether there are MAP kinases in ethylene signalling, either downstream of CTR1 or acting in alternative pathways. Perhaps CTR1 can directly regulate EIN2. The role of EIN2's association with the ethylene receptors is also intriguing. Further work is also required to identify additional players in the pathway, such as the unknown proteins in the high-molecular-mass receptor complexes and in possible bypass pathways. Upstream of the receptors, it is unclear how the receptors obtain the copper cofactor from RAN1 and the mechanism of RTE1 has yet to be elucidated.

A more complete picture of ethylene signal transduction will require an understanding of the dynamics of the protein–protein interactions discussed in this review, such as the regulation and ethylene dependence of the receptor–CTR1 and receptor–EIN2 complexes. To facilitate our understanding, crystal structures are needed for most of these proteins. A greater understanding of post-translational protein modifications, as well as the identification of ethylene-responsive protein targets, should come from proteomic studies, which are just beginning to shed light on such questions (Li *et al.* 2009; Chen *et al.* 2011). Such future studies will help to build a fully dynamic model of the ethylene signal transduction pathway.

## Sources of funding

Our research is supported by a grant from the US National Science Foundation to Dr Chang (MCB0923796). Dr Chang is supported in part by the Maryland Agricultural Experiment Station.

## Contributions by the authors

Both authors contributed to a similar extent overall.

## Conflict of interest statement

None declared.
